# Regulatory T-cell therapy in Crohn’s disease: challenges and advances

**DOI:** 10.1136/gutjnl-2019-319850

**Published:** 2020-01-24

**Authors:** Jennie N Clough, Omer S Omer, Scott Tasker, Graham M Lord, Peter M Irving

**Affiliations:** 1 School of Immunology and Microbial Sciences, King's College London, London, UK; 2 NIHR Biomedical Research Centre at Guy's and Saint Thomas' NHS Foundation Trust and King's College, London, UK; 3 Department of Gastroenterology, Guy's and Saint Thomas' Hospitals NHS Trust, London, UK; 4 Division of Transplantation Immunology and Mucosal Biology, King's College London, London, UK; 5 Faculty of Biology, Medicine and Health, The University of Manchester, Manchester, UK

**Keywords:** Crohn's disease, immunology, immunoregulation, intestinal T cells, T lymphocytes

## Abstract

The prevalence of IBD is rising in the Western world. Despite an increasing repertoire of therapeutic targets, a significant proportion of patients suffer chronic morbidity. Studies in mice and humans have highlighted the critical role of regulatory T cells in immune homeostasis, with defects in number and suppressive function of regulatory T cells seen in patients with Crohn’s disease. We review the function of regulatory T cells and the pathways by which they exert immune tolerance in the intestinal mucosa. We explore the principles and challenges of manufacturing a cell therapy, and discuss clinical trial evidence to date for their safety and efficacy in human disease, with particular focus on the development of a regulatory T-cell therapy for Crohn’s disease.

## Introduction

IBD, chiefly comprising Crohn’s disease (CD) and ulcerative colitis (UC), is a chronic inflammatory group of disorders of the GI tract arising from overexuberant innate and adaptive immune responses to environmental factors in genetically susceptible individuals. IBD affects at least 0.5% of the population in the Western world with 1 million sufferers in USA and 2.5 million in Europe.^1^ Global prevalence continues to increase, largely driven by rising numbers of patients in newly industrialised regions including India and Asia.^1^ The burden of disease is significant with 20%–25% of patients experiencing chronic continuous symptoms which contributes to higher rates of unemployment, sick leave and permanent work disability.^2^ Even with an aggressive top-down approach to therapy, the majority of patients fail to achieve prolonged, steroid-free remission and are at particular risk of requiring surgical intervention. Cumulative surgery rates in CD are high in Europe with 30%–50% of patients requiring surgical intervention and up to 20% needing a reoperation 5–10 years from diagnosis.^2^

As our understanding of the pathophysiology of IBD and its socioeconomic impact has evolved, there has been great impetus to identify novel therapeutic targets to add to the existing arsenal of immunomodulators and biologics. These have focused on a variety of areas including targeting lymphocyte trafficking (vedolizumab, ozanimod, anti-MAdCAM1) and activation (anti-IL (interleukin)-6, anti-IL-12/IL-23), modulating intestinal barrier function (phosphatidylcholine), matrix remodelling (STNM-01, matrix metalloproteinase 9 blocker) and manipulation of gut microbiota (faecal microbiota transplant).^3^ An important pathological process increasingly recognised as driving intestinal inflammation and autoimmunity is the loss of immune homeostasis secondary to qualitative or quantitative defects in the regulatory T-cell (Treg) pool.

Tregs are CD4^+^ T cells that characteristically express the high-affinity IL-2 receptor α-chain (CD25) and master transcription factor Forkhead box P-3 (Foxp3) which is essential for their suppressive phenotype and stability.^4–6^ As activated CD4^+^ T cells can upregulate CD25 expression, an additional defining feature of Tregs is the absence of IL-7 receptor α-chain (CD127).^7^ Their primary function is as dominant controllers of self-tolerance, tissue inflammation and long-term immune homeostasis. Despite making up only 5%–10% of the peripheral CD4^+^ T-cell pool, Tregs exert powerful inhibitory effects on effector cells through a variety of mechanisms including cytokine secretion, metabolic disruption, inhibition of dendritic cells (DCs) and cytolysis. These mechanisms have been rigorously examined using animal models and shown to protect against the development of intestinal inflammation. Studies in patients with IBD have identified defects in the number and distribution of Tregs, and their ability to traffic to the GI tract.^8^ Additionally, resistance to Treg-mediated suppression has been noted in lamina propria T effector cells (Teffs).^9^ These factors are likely to be pivotal in driving intestinal inflammation.

There is growing interest in the therapeutic potential of adoptively transferring healthy Tregs into patients with a wide range of conditions, including IBD and autoimmune disease, in an attempt to shift the balance in areas of active inflammation toward a more tolerogenic microenvironment. Early phase clinical trials have already reported in the fields of solid organ transplantation, graft-versus-host disease (GvHD) and type 1 diabetes mellitus (T1DM) with reassuring safety data and potential signals of efficacy.

This review provides a summary of the suppressive mechanisms used by Tregs and highlights seminal work linking intestinal inflammation with loss of Treg function in both animal models of disease and humans. Additionally, we review ongoing clinical trials with Treg therapy and outline an entirely novel therapeutic strategy for CD using Tregs expanded under good manufacturing practice (GMP) conditions that will be adoptively transferred to patients in an attempt to ameliorate intestinal inflammation and restore immune homeostasis.

## Tregs in health and disease

Tregs can be broadly divided into two groups, thymic Tregs (tTregs) or peripherally induced Tregs (pTregs), based on their developmental origin. Tregs generated in the thymus (tTregs) in the early neonatal period migrate to peripheral organs where they maintain tolerance. This was discovered in 1969 by Nishizuka and Sakakura who showed that in mice, thymectomy 3 days after birth led to the depletion of Foxp3^+^ Tregs and development of autoimmune oophoritis.^10^ In contrast, mice who had thymectomy at day 7 remained healthy as the tTregs had already migrated to the periphery by this point.^11^ Over a decade later, Sakaguchi *et al* demonstrated that day-3 thymectomy autoimmune oophoritis could be prevented with CD4^+^ T-cell inoculation from healthy syngeneic donors. Conversely, the adoptive transfer of T cells from these sick mice was capable of inducing autoimmune disease in healthy T-cell-deficient mice.^11^ Similar findings were noted in rats that underwent adult thymectomy and irradiation resulting in lymphopenia, autoimmune diabetes and insulitis. An injection of CD45RC(low) T cells from healthy donors was capable of preventing disease.^12^ Mottet *et al* subsequently described CD25-expressing CD4^+^ T cells that were able to cure established T-cell transfer colitis.^13^ By the early 2000s, it was clear that a thymically derived CD4^+^CD25^+^ T-cell population possessed the ability to suppress autoreactive T cells and eliminate autoimmunity.

pTregs were first described in 2003 where naive CD4^+^CD25^-^ T cells could be converted into Foxp3-expressing CD4^+^CD25^+^ Tregs by T-cell receptor (TCR) costimulation in the presence of transforming growth factor β (TGF-β).^14^ pTreg conversion in gut-associated lymphoid tissues (GALTs) was enhanced when naive CD4^+^ T cells encountered antigen in the presence of TGF-β, IL-2 and retinoic acid (RA).^15 16^ This is facilitated by CD103^+^ DCs conditioned by the intestinal microenvironment to produce or activate TGF-β and provide RA.^17 18^ In the absence of CD103 expression, DCs fail to induce Treg development and produce proinflammatory cytokines.^17 19^ Additionally, in patients with UC, CD103^+^ DCs appear to have impaired ability to generate pTregs, but induce colitogenic T helper (Th) 1, Th2 and Th17 responses suggesting CD103^+^ DC-mediated pTreg induction is functionally relevant in IBD pathogenesis.^20^

Distinguishing tTregs from pTregs can be difficult as no definitive markers exist. Recently, the expression of the membrane protein neuropilin-1 and the transcription factor Helios by tTregs but not by pTregs has been used to differentiate Treg subsets.^21^ The significance of this lies in the epigenetic differences in the *Foxp3* locus rendering pTregs less stable and more likely to demonstrate plasticity toward a Th17 cell phenotype under inflammatory conditions.^16^ The developmental origin of Tregs selected for expansion as a cell therapy product is therefore an important consideration and will be addressed in more detail later in this review.

The first study identifying Tregs in humans was published in 2001. Baecher-Allan **et al** characterised CD4^+^CD25^+^ T cells in the thymus and peripheral blood which exhibited anti-inflammatory and suppressive properties.^22^ Subsequent work established Foxp3 as the master transcription factor for Tregs.^4 6 23^ Foxp3 can however be expressed transiently in non-regulatory CD4^+^ T cells on TCR activation and the CD4^+^CD25^+^CD127^lo^ surface phenotype must be used to define Tregs.^24^ Inactivating mutations in *Foxp3* clinically manifest as severe autoimmunity with a scurfy phenotype in mice and IPEX syndrome (immune dysregulation, polyendocrinopathy, enteropathy, X-linked) in humans.^25–28^ With autoimmune enteropathy (manifesting as chronic diarrhoea and malabsorption) a predominant feature, attention was focused on the functional role of Tregs within the GI tract.

pTregs are found in abundance in the intestinal lamina propria where interactions with environmental antigens can shape phenotypic differences and transcription factor expression.^29^ The gut microbiota represents a substantial antigen load driving the expansion of colonic pTregs that coexpress the Th17 master transcription factor RORγt.^30^ These Foxp3^+^ RORγt^+^ pTregs have a stable regulatory phenotype and provide tolerance towards the gut microbiota.^31 32^ Conversely, RORγt^-^ pTregs are found in the small intestine where they are induced by dietary antigens and repress underlying Th1 cell responses to ingested proteins.^33^ Finally, an intestinal tTreg population that coexpress the Th2 master transcription factor, GATA3, has been shown to mediate repair of the intestinal mucosa. GATA3^+^ tTregs express high levels of the IL-33 receptor, ST2, and amphiregulin (AREG), an epidermal growth factor receptor ligand involved in tissue repair.^34 35^

Following on from the fundamental observations linking Treg dysfunction to an array of autoimmune polyendocrine syndromes, studies began to emerge identifying defects in either number or function of peripheral blood Tregs in autoimmune disorders including IBD, T1DM, multiple sclerosis, systemic lupus erythematosus (SLE), myasthenia gravis and rheumatoid arthritis.^8 36–40^ Maul *et al* observed that in patients with active IBD, the intestinal lamina propria Treg pool was significantly smaller than that of a positive control, namely diverticulitis.^8^ Additionally, in these patients, the peripheral blood Treg pool was smaller than that of inactive IBD or diverticulitis.^8^ Interestingly, the peripheral blood Tregs retained their suppressive capacity suggesting that disease may be driven by ineffective trafficking to the gut and reduced numbers of Tregs. Furthermore, colitogenic T cells from patients with IBD appear to be resistant to TGF-β1-mediated Treg suppression highlighting an additional defect in immunological tolerance that may drive disease.^9^

## Treg function and colitis

**Figure 1:**
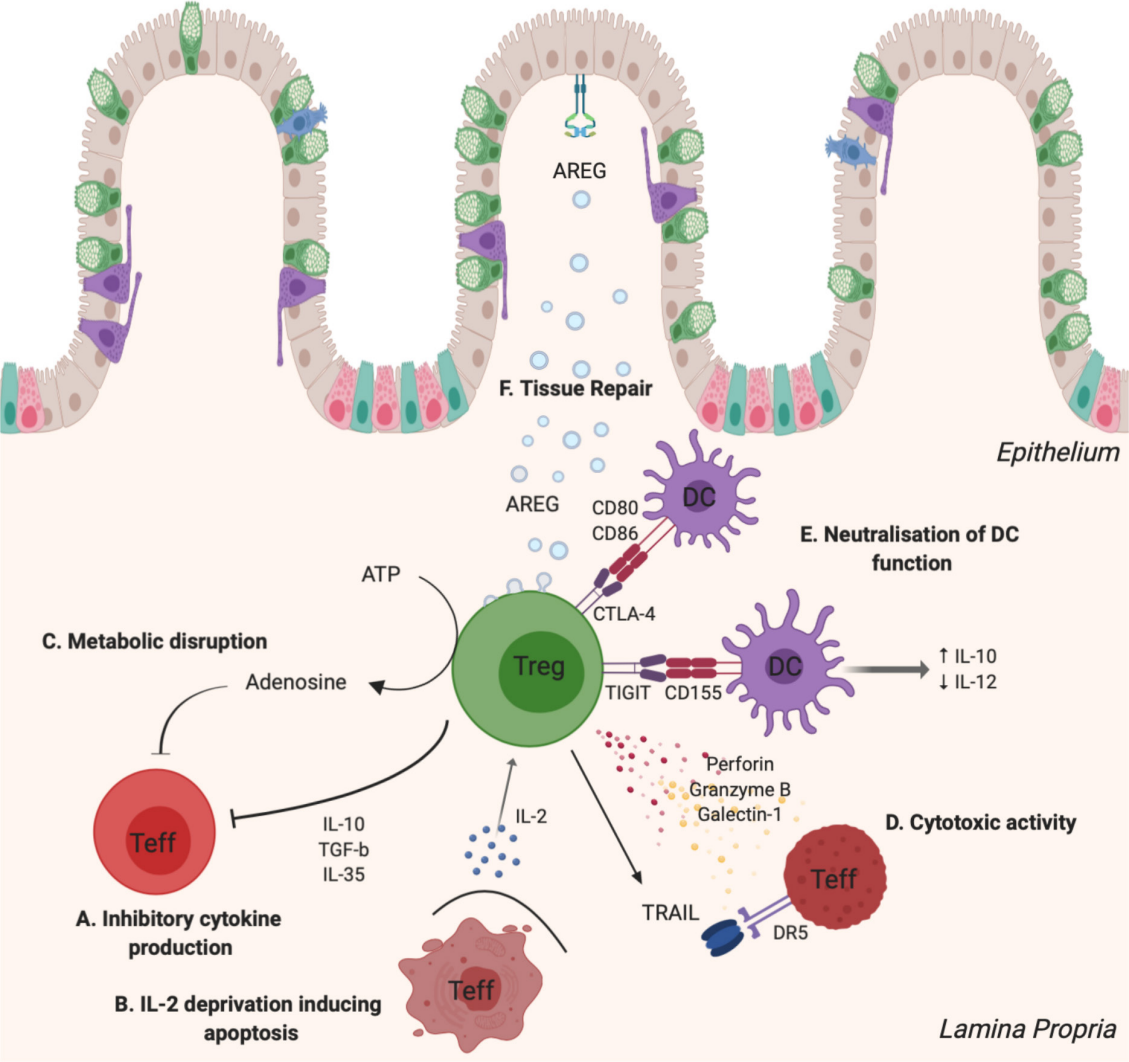
Mechanisms of Treg-mediated suppression. Tregs use a multitude of mechanisms to promote a tolerogenic microenvironment and tissue repair. (A) Secretion of the anti-inflammatory cytokines, IL-10, TGF-β and IL-35, not only inhibit Teff proliferation but also suppress Th1 and Th17 effector function, both of which are key mediators of IBD. (B) Tregs express the high-affinity IL-2 receptor α-chain (CD25) consuming local IL-2 with greater affinity than effector cells. Teffs which are ‘starved’ of IL-2 exhibit restricted proliferation and undergo apoptosis. (C) Tregs coexpressing CD39 and CD73 disrupt metabolic processes in effector cells by converting ATP into pericellular adenosine, a potent inhibitor of Teff function. Additionally, adenosine stimulates TGF-β production, promoting development of pTregs. (D) Tregs are capable of secreting perforin, granzyme B and galectin-1 which are directly cytotoxic against Teffs. Activated Tregs also express TRAIL, inducing apoptosis of Teffs through the TRAIL/DR5 pathway. (E) Expression of CTLA-4 degrades DC-derived CD80 and CD86 leading to impaired CD28-mediated costimulation of T cells. DC function is further inhibited through the interaction of Treg-derived TIGIT and CD155 on DCs. This induces IL-10 production and suppresses IL-12. (F) In response to alarmins, Tregs produce AREG, an important regulator of tissue repair and regeneration. AREG, amphiregulin; CTLA-4, cytotoxic T lymphocyte associated protein 4; DC, dendritic cell; DR5, death receptor 5; IL, interleukin; pTregs, peripheral regulatory T cells; Teff, T effector lymphocyte; TGF-β, transforming growth factor beta; Th1, T helper 1 cell; Th17, T helper 17 cell; TIGIT, T-cell immunoreceptor with Ig and immunoreceptor tyrosine-based inhibitory motif domains; TRAIL, TNF-related apoptosis-inducing ligand; Treg, regulatory T cell. Figure generated using BioRender illustration software.

Tregs function as key mediators of peripheral tolerance through direct cellular contact and paracrine actions on tissues where they reside.^41 42^ It is essential that Tregs effectively traffic to target organs where they promote a tolerogenic microenvironment. An important example is IL-10-secreting Tregs that reside in the GI mucosa and control inflammatory responses induced by environmental insults. Selective disruption of IL-10 expression in these Tregs has been shown to cause spontaneous colitis.^43^ This is one of many modalities that Tregs can employ to maintain immune homeostasis at the mucosal interface. Others include inhibitory cytokine secretion, cytolysis of effector cells, metabolic disruption, neutralisation of antigen presenting cells and promotion of tissue repair.^44^ These functions will be reviewed in further detail outlining their associations with intestinal inflammation (see figure 1).

### Inhibitory cytokines

The Treg cytokine repertoire includes the anti-inflammatory molecules IL-10, TGF-β and IL-35. The expression of IL-10 and IL-35 requires TCR signalling, suggesting that Treg function in part relies on antigen encounter in the local microenvironment.^45^ Pioneering work by Powrie *et al* over 20 years ago showcased the potent inhibitory ability of IL-10, where recombinant IL-10 therapy ameliorated established T-cell transfer colitis.^46^ Subsequently, the cotransfer of CD45RB(low) T cells were shown to prevent colitis and IL-10 was identified as an essential mediator for this *in vivo* suppression.^47^ The suppressive effects of Treg-derived IL-10 in mice appear to be specific for mucosal surfaces rather than controlling systemic autoimmunity.^43^ Further studies have demonstrated that IL-10 induces robust activation of a STAT3 (signal transducer and activator of transcription 3)-dependent Th17 suppression programme in Tregs, downstream of IL-10R.^48^ This suppresses pathogenic Th17 cell responses and ablation of IL-10R in Tregs has been shown to cause colitis. It is therefore plausible that disordered IL-10 signalling may contribute to aberrant Th17 activity, which is implicated in IBD.^49^ In fact, there have been several cases of homozygous loss-of-function mutations in *Il-10* and *Il-10r* arising in individuals from consanguineous marriages. These resulted in infantile severe, progressive, intractable Crohn’s-like colitis.^50^

TGF-β plays an important role inducing pTreg formation on antigen encounter in GALT and has a functional role in suppressing proinflammatory pathways.^15^ Tregs are capable of producing TGF-β, which profoundly suppresses the proliferation of Teffs.^51^ Treg-derived TGF-β1 inhibits Th1-cell differentiation and IBD in a transfer model of colitis.^52^ Conversely, Tregs from TGF-β1-deficient mice fail to suppress intestinal inflammation in a severe combined immunodeficiency (SCID) transfer model of colitis.^51^ Human studies have supported these early findings; a study on healthy human colonic biopsies and lamina propria mononuclear cells (LPMCs) treated with anti-TGF-β neutralising antibody showed that TGF-β is a critical suppressor of T-bet (T-box transcription factor)-dependent Teff proliferation and Th1 cytokine expression.^53^ This suggests a role for TGF-β in suppressing intestinal inflammation in humans. Indeed, MacDonald *et al* have shown that colonic tissue and isolated T cells from patients with CD overexpress Smad7, an inhibitor of TGF-β1 signalling.^54^ Furthermore, colonic LPMCs from patients with CD were resistant to Treg-mediated suppression, a phenomenon that could be reversed with Smad7 antisense treatment.^9^ Smad 7 antisense therapy (Mongersen) was subsequently evaluated in CD but, despite promising early phase data, a phase III clinical trial was terminated early due to the lack of benefit.^55 56^ Although Mongersen may overcome Teff resistance to TGF-β, it is possible that in CD there are insufficient numbers of functional Tregs in the mucosal environment to produce TGF-β explaining the disappointing trial outcome.

IL-35 is a heterodimer of Epstein-Barr virus-induced 3 (Ebi3) and IL-12α that is constitutively expressed in Foxp3^+^ Tregs but not Teffs. It was first described in 2007 where *Ebi3^-/-^* and *IL-12α ^-/-^* Tregs were shown to have significantly reduced regulatory activity *in vitro* and failed to cure T-cell transfer colitis *in vivo*.^57^ Additionally, IL-35 can induce the generation of a regulatory population from naive mouse or human CD4^+^ T cells. These so-called iT(R)35 cells mediate suppression via IL-35 alone, do not express Foxp3 and are strongly suppressive and stable *in vivo*.^58^ In both dextran sulfate sodium and 2,4,6-trinitrobenzene sulfonic acid colitis, recombinant IL-35 therapy can treat disease through downregulation of the Th1 and Th17 master transcription factors, T-bet and RORC (related orphan receptor C), respectively, and through inhibition of IFN-γ, IL-6 and IL-17.^59^

### Inhibition of metabolic processes

While Tregs are not known to produce IL-2, their development and function is critically dependent on this cytokine. IL-2 and the transcription factor STAT5, downstream of IL-2 receptor (IL-2R), induce the expression of Foxp3 and differentiation of tTregs.^60^ Furthermore, STAT5 activation driven by IL-2R signalling enhances the suppressor function of differentiated Tregs.^61^ An absence of IL-2 signalling has been shown to reduce the number and functional activity of Tregs, predisposing to autoimmunity and inflammation.^62 63^ The structural conformation of IL-2R in Tregs provides a competitive advantage for IL-2-receptor engagement over alternative cell subsets. Tregs abundantly express IL-2 receptor α-chain (CD25), which together with the common γ-chain (γc, CD132) and IL-2 receptor β-chain (CD122) form a characteristic three-subunit receptor configuration. This confers a~1000 fold increase in receptor affinity for IL-2 over Teffs.^64^ In a proinflammatory environment dominated by actively dividing effector cells, Tregs have the ability to ‘consume’ local IL-2, starving effector cells of this essential cytokine for survival and proliferation.^42 65^ Moreover, this mechanism has been shown to induce the apoptosis of effector cells.^66^ This highlights an important TCR-independent paracrine mode of suppression in local tissues, facilitated through the constitutive expression of high-affinity IL-2R (containing CD25). There has been a handful of cases of CD25 deficiency in humans often manifesting in an IPEX-like syndrome.^67–69^ A notable case which presented with autoimmune enteropathy at 6 months had Foxp3^+^ Tregs with defective IL-10 expression suggesting that IL-2 responsiveness is important for Treg-mediated IL-10 production.

Tregs can also interfere with ATP metabolism to dampen proinflammatory responses. Tregs coexpress the ectoenzymes CD39 and CD73 responsible for the degradation of ATP and generation of pericellular adenosine.^70^ Adenosine stimulates the A2A receptor on Teffs exerting potent inhibitory effects. Activation of the A2A receptor also inhibits IL-6 expression while enhancing the production of TGF-β.^71^ This promotes the development of adaptive induced Tregs and simultaneously inhibits proinflammatory Th17 cell formation. Furthermore, signalling through the A2A receptor appears to control *in vivo* murine colitis.^72^

### Neutralisation of dendritic cell function

The activation of T cells requires TCR antigen/major histocompatibility complex engagement in the context of a secondary signal, namely T-cell-derived CD28 binding the DC B7 ligands, CD80 and CD86. This process is negatively regulated through the production of cytotoxic T lymphocyte associated protein 4 (CTLA-4) which is constitutively expressed in Foxp3^+^ Tregs.^73^ CTLA-4-expressing cells can capture CD80 and CD86 by a process of trans-endocytosis and degrade these ligands, resulting in impaired costimulation via CD28.^74^ This is a functionally significant process with Treg-conditioned DCs inducing poor T-cell proliferation.^75^ An additional mechanism mediated through the interaction of CTLA-4 and CD80/CD86 is the upregulation of indoleamine 2, 3-deoxygenase in DCs. This is a potent regulatory molecule which catabolises the essential amino acid tryptophan to the proapoptotic metabolite kynurenine leading to the suppression of Teff function.^60^ *In vivo* models have demonstrated that CTLA-4 is essential in preventing autoimmunity. Selective deletion of CTLA-4 in Tregs of BALB/c mice results in fatal T-cell-mediated autoimmune disease at just 20 days of age.^76^ Additionally, several cases of germline heterozygous mutations in CTLA-4 have been identified in humans.^77^ CTLA-4 haploinsufficiency resulted in dysregulation of Tregs, hyperactivation of Teffs and lymphocytic infiltration of target organs including the GI tract. It was recently discovered that lipopolysaccharide-responsive and beige-like anchor protein (LRBA) regulates CTLA-4 expression, where mutations in LRBA lead to reduced levels of CTLA-4.^78^ These mutations are commonly associated with primary immunodeficiency, reduced Treg numbers and susceptibility to IBD.^79 80^

Recently, the coinhibitory molecule T-cell immunoreceptor withimmunoglobulin and immunoreceptor tyrosine-based inhibitory motif (ITIM) domains (TIGIT) has been described as an inhibitor of autoimmune responses through its interactions with DCs and T cells. TIGIT interacts with its ligand CD155 on DCs to induce IL-10 and suppress IL-12 production, thereby inhibiting Th1 responses.^81^ As Tregs are the primary cell type that constitutively express TIGIT, it has been suggested that the observed effects on DCs are mediated by TIGIT^+^ Tregs. Furthermore, Tregs expressing TIGIT have been shown to directly suppress Th1 and Th17 responses through the production of the effector molecule fibrinogen-like protein 2.^82^

### Cytotoxic activity

Historically, cytotoxic activity has been associated with natural killer cells and cytotoxic T lymphocytes (CD8^+^ T cells). In 2004, Grossman *et al* first described granzyme-B expressing CD4^+^ Tregs capable of killing target cells in a perforin-dependent, but TCR-independent manner.^83^ Boissonnas *et al* subsequently showed that in a mouse tumour model, Foxp3^+^ T cells can kill antigen-specific DCs. Treg cytotoxicity has also been observed against CD4^+^ T cells in both *in vitro* and *in vivo* models. Activated Tregs upregulate tumour necrosis factor-related apoptosis inducing ligand (TRAIL) which enhances suppressive activity as well as cytotoxicity against CD4^+^ T cells. This is entirely dependent on the TRAIL/death receptor 5 pathway.^84^ Galectin-1, a β-galactoside-binding protein known to induce T-cell apoptosis has also been implicated in Treg cytotoxic function. Galectin-1 was found to be overexpressed in Tregs and galectin-1 knockout models were shown to possess reduced regulatory activity.^85^

### Tissue repair

Aside from limiting mucosal damage through the suppression of proinflammatory cells following environmental insults like infection, Tregs may also promote tissue repair. Recently, the epidermal growth factor-like molecule AREG has gained attention as an important regulator of tissue repair and regeneration. In a murine model of influenza, selective Treg deficiency in AREG leads to severe acute lung damage without any alterations in Treg suppressor function. This suggests that Tregs play a direct role in tissue repair and maintenance that is distinct from their suppressive function.^86^ Treg production of AREG is dependent on IL-18 or IL-33 which function as endogenous danger signals or alarmins, in response to tissue damage.^86^ Studies in humans have revealed high levels of IL-33 in inflamed lesions of patients with IBD, and Tregs expressing the IL-33 receptor, ST2, are enriched in the colon.^35 87 88^ IL-33-Treg signalling may therefore represent an important pathway in both disease pathogenesis and recovery.

## Tregs as a therapeutic product

In light of the vast array of preclinical data showcasing how a multitude of defects in Treg function contribute to autoimmunity and inflammation, including IBD, there has been great interest in harnessing the suppressive ability of Tregs as a therapeutic product. Consequently, there are over 50 registered trials of Treg therapy that are either completed or ongoing (ClinicalTrials.gov). Most of these trials involve adoptive cell transfer, although the dose of Tregs given is highly variable. In the setting of autoimmune disease and transplantation, the goals of treatment are the restoration of peripheral self-tolerance, the suppression of inflammation and promotion of tissue repair.^89^

To become a successful therapeutic product, Tregs must home to sites of inflammation and secondary lymphoid tissues and must undergo TCR engagement. It has been demonstrated in solid organ transplantation that alloantigen-specific Tregs provide higher therapeutic benefits than polyclonal Tregs, without delivering a systemic immunosuppressive effect.^90^ Directing Tregs against a specific alloantigen also permits immunomodulatory functions to be concentrated at the site of the alloantigen source, circumventing the relative paucity of Tregs. An early study demonstrated that peripheral Treg expansion in mice could be driven by prolonged low-dose subcutaneous infusion of a specific peptide.^91^ The induced Tregs had suppressive abilities and demonstrated high levels of Foxp3 expression indicating a stable Treg phenotype. However, in IBD, a specific antigen has yet to be identified.

The relative paucity of Tregs in peripheral blood represents an obstacle to the development of a cellular therapy, though the optimum number of Tregs to be infused remains unclear. It has been suggested that the number of Tregs given should be at least as great as the number of Teffs in the body,^92^ though Tregs also exhibit the ability to confer suppressive ability on conventional T cells through ‘infectious tolerance’.^90^ In this process, the direct secretion of TGF-β, IL-10 and IL-35 by Tregs, and indirect induction via DCs, can generate a regulatory microenvironment which may partially circumvent the problem of low absolute numbers of Tregs.^93^

Several groups have developed protocols in line with GMP requirements to permit *ex vivo* cell expansion of Tregs.^92 94 95^ GMP-manufactured Tregs delivered in some early trials were only around 50% pure, but the development of plastic beads coated with stimulatory antibodies and the discovery of additional surface markers for Treg phenotyping mean that a product with purity greater than 90% is now achievable.^92^ Contamination of the expansion product with Teffs hampers expansion,^96^ but the inclusion of rapamycin in cell culture blocks expansion of Teffs without affecting Treg proliferation, leading to the preferential promotion of Treg proliferation.^92 97^

Tregs are first isolated from peripheral blood by surface marker expression (CD4^+^CD25^hi^CD127^lo^). This can be performed using stream in air fluorescence-activated cell sorters (FACS) which yield a highly pure starting population, but the necessary air exposure requires high-efficiency particulate air (HEPA) enclosures, and single-use sample lines to be compatible with manufacturing GMP cell products. Closed system magnetic bead-activated cell sorting (MACS) can be adapted for large-scale isolation of human Tregs, but unlike FACS cannot easily distinguish surface marker expression density. A recently developed microfluidic chip FACS, the MACSQuant Tyto (Miltenyi Biotech, Germany) surmounts the problems of stream in air sorters, as the cells remain in a closed system throughout the sorting process. Expansion of the sorted cells is achieved through polyclonal TCR activation with anti-CD3/anti-CD28 beads.^98^ Tregs are sampled and checked for sterility and phenotype throughout the expansion process. With optimised conditions, a 500-fold expansion can be anticipated over a 14-day period.^95^

Uncertainty about the plasticity of Tregs in culture and following infusion means there is a theoretical concern about the development of a proinflammatory phenotype, which could lead to transplant rejection or aggravation of inflammation. However, rapamycin-expanded Tregs are not contaminated by IL-17-producing Th17 cells, and these cells maintain a stable phenotype on transfer in vivo to mice.^99^ Canavan **et al** found that the starting population for Treg expansion from the peripheral blood of patients with CD has a critical effect on the phenotype of the expanded cell population.^94^ Tregs from a highly pure FACS-sorted ‘naive’ CD4^+^CD25^hi^CD127^lo^CD45RA^+^ precursor population demonstrated enhanced suppressive ability and reduced Th17 plasticity in vitro compared with a FACS-sorted CD4^+^CD25^hi^CD127^lo^CD45RA^-^ or MACS-enriched CD8^-^CD25^+^ population. Rapamycin appears to imprint a fixed CD4^+^CD25^hi^ phenotype to cells expanded from a ‘naive’ CD45RA^+^ population, as evidenced by the retention of demethylation at the Foxp3 locus.

## Treg therapy in other conditions

There is an increasing body of evidence for the use of Tregs as cellular therapy in autoimmune disease and transplantation (see table 1). Adoptive transfer of Tregs to prevent GvHD was the first illustration of the potent therapeutic potential of Tregs in experimental transplantation.

**Table 1:** Summary of ClinicalTrials.gov listings for reported trials using in vitro expanded regulatory T-cell (Treg) therapy

Study	Clinical context	Enrichment protocol	Expansion protocol	Dose	Study outcome
Trzonkowski **et al** 96	Treatment of acute and chronic GvHD (n=2)	Tregs from allogenic buffy coat. CD4^+^ negative bead selection followed by FACS-based sorting of CD4^+^CD25^hi^CD127^lo^ cells	RPMI 1640 with 10% autologous plasma IL-2 (1000 IU/mL) Anti-CD3/anti-CD28 beads (1:1) 3 weeks	Acute GvHD: 1×10^6^/kg Chronic GvHD: 3×10^6^/kg	Transient improvement in acute GvHD; alleviation of symptoms and reduction of immunosuppression in chronic GvHD
Brunstein *et al* 151	Prevention of GvHD following umbilical cord blood transplantation (n=23)	CD25^+^ bead-positive selection	X-Vivo 15 with 10% human AB serum IL-2 (300 IU/mL) Anti-CD3/anti-CD28 beads (1:2) 18±1 days	0.1−30×10^5^/kg	Well-tolerated; reduced incidence of grade II–IV GvHD in Treg recipients
Marek-Trzonkowska *et al* 118	Safety of autologous in vitro expanded Tregs in paediatric type 1 diabetes (n=10)	FACS-based sorting of CD3^+^CD4^+^CD25^hi^CD127^lo^ cells	CellGro medium with 10% autologous plasma IL-2 (1000 IU/mL) Anti-CD3/anti-CD28 beads (1:1) Up to 2 weeks	10−20×10^6^/kg	Well-tolerated; decreased insulin requirements and C-peptide levels in Treg recipients
Desreumaux **et al** 139	Safety and efficacy in Crohn’s disease (n=20)	Culture of PBMCs with ovalbumin, IL-2 and IL-4 followed by cloning of ovalbumin-specific T cells	X-Vivo 15 IL-2 (200 IU/mL) Anti-CD3/anti-CD28 beads (1:1) Ova-Tregs selected based on ovalbumin-specific IL-10 production 12 to 15 weeks	1×10^6^–1×10^9^	Well-tolerated; dose-related efficacy
Bluestone **et al** 95	Safety in adults with type 1 diabetes (n=14)	FACS-based sorting of CD4^+^CD25^hi^CD127^lo^ cells	X-Vivo 15 with 10% human AB serum and deuterated glucose IL-2 (300 IU/mL) Anti-CD3/anti-CD28 beads (1:1) 14 days	0.05×10^8^–26×10^8^	Well-tolerated, no significant adverse events. Stable C-peptide levels and insulin use in recipients for up to 2 years postinfusion
Mathew **et al** 108	Safety in living donor kidney transplant (n=9)	CliniMACS plus GMP enrichment system (Miltenyi)	IL-2 (1000 IU/mL) MACS GMP expansion beads 1:1-4:1 3 weeks	0.5−5×10^9^	Well-tolerated, no infections or rejection up to 2 years post-transplant

FACS, fluorescence-activated cell sorting; GMP, good manufacturing practice; GvHD, graft-versus-host disease; IL, interleukin; MACS, magnetic bead-activated cell sorting; PBMC, peripheral blood mononuclear cell; RPMI 1640, Roswell Park Memorial Institute 1640 medium; Treg, regulatory T cell.

### Graft-versus-host disease

The risk of developing GvHD following haematopoetic stem cell transplantation (HSCT) is associated with low numbers of Tregs in the periphery,^100^ and in vivo expansion of Tregs post-HSCT using low-dose IL-2 has demonstrated efficacy against GvHD.^101 102^ Studies in mice involving infusion of cultured CD4^+^CD25^+^ T cells resulted in a significantly reduced GvHD phenotype,^103^ and in humans it was found that infusion of freshly isolated donor Tregs given at the same time as haplotype mismatched HSCT prevented the development of GvHD.^104^

Five trials of *ex vivo* expanded Tregs have to date involved small numbers of patients only, but suggest therapy can prevent or delay the onset of chronic GvHD.^105 106^ Treg therapy seems to be effective only in the chronic form of GvHD, but this may be because of the time requirements to expand the cellular product which makes it difficult to administer in a timely manner in acute GvHD.^107^

### Solid organ transplant

Adoptive Treg therapy has been trialled following renal and liver transplantation, with the aim of inducing tolerance to the allograft and reducing the burden of long-term immunosuppression.^108^ Tregs have been shown to control immune responsiveness to alloantigens and contribute to ‘operational tolerance’ in preclinical transplantation models.^109 110^ Recipient-derived Tregs expanded for direct and indirect pathway allospecificity *in vitro* were able to mediate effective protection against acute and chronic rejection in skin and heart allografts in mice^111^ and could be used to induce tolerance of a murine skin transplant following thymectomy and T-cell depletion.^112^ In these models, alloantigen reactive Tregs were more effective at preventing graft rejection than polyclonally expanded Tregs.^98^

A phase I study in renal transplantation recruited nine living donor transplant recipients and used the product of leukapharesis as the basis for *ex vivo* expansion of polyclonal autologous Tregs.^108^ Alemtuzumab was given at induction to achieve lymphodepletion, on the basis of previous experiments suggesting a reduction in circulating Teffs worked synergistically with Treg infusion to prolong allograft survival.^110^ Recipients were switched from traditional immunosuppression with tacrolimus, which blocks IL-2 production, to sirolimus (rapamycin), which has Treg promoting activity.^113^

An enhanced suppressive ability of the expanded Tregs was demonstrated when compared with Tregs taken directly *ex vivo*.^108^ There were no adverse infusion-related side effects, infections or rejection up to 2 years post-transplant, and there was a 5–20 fold increase in the number of circulating Tregs seen up to 1 year post-transplant. Transplant biopsies taken at 3 months did not show rejection and recipients had not developed peripheral donor-specific antibodies. An additional important outcome from trials in transplantation is that they have demonstrated that it is possible to expand Tregs from immunocompromised patients.^114^

A trial of Treg immunotherapy in liver transplantation is currently underway.^115^ This is predicated on the observation that when liver allografts in mice were infiltrated with Tregs, loss of Treg numbers was associated with a loss of tolerance.^116^ Increased frequencies of Tregs are also seen in human subjects who acquire ‘operational tolerance’ to their liver transplant.^117^

### Type 1 diabetes mellitus

The development of T1DM is associated with deficits in the number and suppressive activity of Tregs.^118^ Accelerated diabetes onset is seen in both scurfy mice^27^ and children with IPEX,^119^ highlighting the role of Tregs in protecting pancreatic islet cells from destruction. Tregs have been implicated in the pathogenesis of diabetes in the non-obese diabetic mouse model,^120 121^ and anti-CD3 antibodies have been efficacious in the treatment of diabetes in both mouse^122 123^ and human trials.^124 125^ Subjects exhibited lower insulin requirements and higher C-peptide levels at least 18 months after a short course of intravenous treatment, with evidence of anti-CD3 treatment inducing expansion of a CD4^+^CD25^+^ T-cell population.^123^A trial of 10 children treated with expanded polyclonal Tregs within 2 months of their diagnosis demonstrated statistically lower insulin requirements and C peptide levels compared with matched controls up to 6 months postinfusion, with two patients remaining insulin-independent.^118^ There were no serious adverse events up to 1 year following infusion.

In a phase I open-label trial of 14 adult patients infused with *ex vivo* expanded Tregs in escalating doses, 7 of 14 patients had stable C peptide levels and insulin use for up to 2 years following infusion.^95^ However, the study was not powered to detect significant clinical improvement. There were no infusion reactions or therapy-related serious adverse events. Phenotypic analysis of the cell product after expansion and after infusion identified stable surface marker expression, demonstrating that the infused Tregs did not acquire a pathological phenotype. High-throughput TCR-β sequencing analysis indicated that expanded Tregs retained a high degree of diversity.

Adoptively transferred Tregs were tagged by labelling the deoxyribose moiety of replicating DNA during expansion *ex vivo*, through the addition of deuterated [6,6-^2^H_2_] glucose to Treg culture throughout the 14-day expansion period.^95^ Patient samples were analysed by gas chromatography–mass spectrometry for deuterium enrichment to create pharmacokinetic curves. Adoptively transferred T-cell numbers peaked at 2 weeks following infusion, but were still detectable at up to 25% of the peak level at 1 year in peripheral blood. Significantly, deuterium labelling was never found in non-Tregs, indicating the stability of infused Tregs. However, due to the nature of this study, the stability of these cells was not assessed within the target tissue.

## Treg therapy in IBD

A local imbalance between Treg and Teff responses plays a key role in the development of gut inflammation in IBD.^8^ T-cell gut homing is mediated by specific interaction between integrin α4β7 and its ligand mucosal vascular addressin cell adhesion molecule 1 (MAdCAM-1).^126 127^ Several groups have shown that transfer of Tregs into mice leads to clinical and histological improvement in colitis,^13 128 129^ and rapamycin-expanded Tregs ameliorated established colitis in an SCID mouse model.^130^ Polyclonality of the TCR is likely to be an important requirement for Tregs to maintain intestinal homeostasis in vivo. Mice which express a restricted TCR repertoire develop spontaneous colitis due to a loss of tolerance to intestinal microbiota.^131^

Several groups have demonstrated that it is feasible to extract Tregs from patients, and expand them in vitro under GMP conditions, including from subjects receiving thiopurines and anti-TNFα medications.^92 94 97 132^ Even after prolonged culture, these Tregs maintained Foxp3 expression and demonstrated enhanced suppression of autologous T cells. Uncertainty regarding the potential for adoptively transferred Tregs to express IL-17 and exacerbate CD lesions is a concern. However, the administration of proinflammatory cytokines (IL-1, IL-2, IL-6, IL-21, IL-21 and TGF-β) failed to induce IL-17 production by CD45RA^+^ expanded Tregs in vitro.^94^

### Antigen specific versus polyclonal Treg cell products for CD

No antigens have yet been verified as causal in CD. Attempts have been made to identify shared TCRs between CD sufferers with the aim of discovering target antigens.^133–135^ This work has observed that the CD4 TCR repertoires are significantly more diverse in patients with CD and UC than healthy controls. This may be explained by GI barrier disruption increasing the number of antigen presentation events in comparison to a healthy gut. Resolving a target from the GI peptidome is challenging due to the heterogeneous nature of the environment. Developments in the understanding of non-conventional epitopes are also increasing the magnitude and complexity of the peptidome itself.^136^ In the absence of a known target, the broad reactivity of a polyclonal Treg product may be advantageous, as the cell product will recognise millions of putative epitopes, increasing the likelihood of TCR engagement and subsequent Treg activation. Sequencing of isolated Tregs from GI biopsies post-transfer may yield novel targets, on which chimeric antigen receptor technology could be readily implemented.^137^

For Treg therapy to be effective in IBD, expanded Tregs must have the ability to home to the gut.^138^ A French group reported the results of an open-label multicentre phase I/IIa trial of ovalbumin-specific Tregs in 20 patients with refractory CD.^139^ Ovalbumin is a common food antigen and is not implicated in intestinal inflammation in animal models or in patients with CD. Its distribution along the digestive tract can be used to activate Tregs locally. In the study, this was facilitated through ingestion of meringue cakes by subjects.^139^

The cell product was cultured in the presence of ovalbumin, and trial subjects received a dose of 10^6^–10^9^ Tregs.^139^ Patients enrolled in the study had at least moderately active CD, with a Crohn’s Disease Activity Index (CDAI) greater than or equal to 220 within 6 months of screening, and a washout period was required for immunosuppression and anti-TNFα therapy. The infusion was well tolerated, with mild GI symptoms and CD flares being the most commonly reported adverse effects. Two patients experienced thrombotic events, but these are known to occur more frequently in inflammatory conditions including active CD.^140^ Eight (40%) patients had a significant CDAI response at weeks 5 and 8 after treatment, with two patients experiencing sustained remission. Overall, the results suggested good tolerability in this disease group with possible signals of efficacy.

In the absence of a known antigen, other methods must be used to direct the Tregs to the areas of inflammation. A recent study has shown that a highly specific retinoic acid receptor α (RARα) agonist induces expression of Integrin α4β7 (the ligand of MAdCAM-1) on the Treg surface. Adoptive transfer of RARα agonist-treated Tregs leads to improved Treg trafficking to gut tissue in a humanised mouse model of colitis.^94^ Supporting this mechanism for resolving inflammation, another group have demonstrated that DCs can be engineered de novo to produce high concentrations of RA.^141^ When transferred to mice, the RA-secreting DCs were able to augment the expression of Foxp3 and the gut-homing receptor CCR9 in native Tregs with the subsequent suppression of colitis.

The RARα agonist treated cell product forms the basis of the TRIBUTE trial (ClinicalTrials.gov Identifier: NCT03185000), a double-blinded placebo-controlled phase I/IIa trial of adoptive Treg therapy in CD.

## Future developments in Treg therapy

The potential therapeutic benefits of adoptive cell therapy are being explored in numerous autoimmune conditions. In SLE, adoptive transfer of *ex vivo* expanded Tregs in mice delayed the onset of renal complications and prolonged survival,^142 143^ and a pilot study of low-dose IL-2 in 37 patients led to increased circulating peripheral Treg numbers and decreased SLE disease activity scores.^144^ Adoptive Treg transfer in a single patient with cutaneous lupus did not lead to clinical benefit, but increased percentages of highly activated Tregs were identified in biopsies taken from diseased skin.^145^ Treg accumulation in skin was associated with a marked attenuation of IFN-γ, which was more pronounced relative to peripheral blood.

Preliminary results from mouse models suggest a role for Treg therapy in conditions as diverse as pemphigus vulgaris,^146^ autoimmune hepatitis,^147^ multiple sclerosis,^107^ asthma and allergy, in which antigen-specific Tregs may represent a viable therapeutic option.^148 149^

Many ongoing challenges exist for the advancement of Treg therapy. Uncertainties remain about the optimal timing of *ex vivo* Treg expansion, and whether IL-2 administration would be a useful adjunct to support a Treg population *in vivo*.^95 101^ In addition, concomitant treatment of autoimmune disease with immunosuppressive drugs may affect the function of adoptively transferred cells.^89^

The optimal dosing strategy for Treg therapy also remains unclear, although data tracking the survival of deuterium-labelled Tregs in vivo could be invaluable in informing a suitable dosing regimen.^95^ A two-phase decay in numbers of deuterium-labelled Tregs has been seen, with 75% of the peak level lost at 3 months. However, levels stabilised at 1 year, with up to 25% of peak Treg numbers remaining in the peripheral circulation. The decrease in labelled Tregs may represent cell death, trafficking to lymphoid tissue and sites of inflammation, or proliferation of the Treg compartment leading to dilution of deuterium enrichment. Reassuringly, at no point during the trial was deuterium detected in cell populations other than Tregs, suggesting a stable phenotype *in vivo*.^95^

Tracking of TCR clonotypes may also provide useful data on Treg kinetics and dispersal. Analysis of the TCR repertoire has suggested that the kinetics of transferred Tregs in peripheral blood varies significantly between individuals.^150^ In a descriptive study, the TCR Vα chain was sequenced in two patients receiving donor Treg infusion.^150^ Treg therapy altered the patients’ peripheral TCR repertoire considerably toward that of the infused cell product, but to different degrees in each patient. Importantly, the degree of alteration of the TCR repertoire appeared to correlate with clinical response. This suggests that monitoring TCR repertoires following adoptive cell transfer may provide clinically meaningful information.

## Conclusion

There is now robust evidence of the therapeutic potential of Treg therapy in CD. Trials in multiple autoimmune diseases and results from use of ovalbumin-specific Tregs in IBD show promising early signs of efficacy. The safety signal is reassuring, with evidence that the adoptively transferred Treg phenotype is stable in vivo. Results from deuterium labelling suggest that infused Tregs may be able to exert a long-lasting systemic effect with labelled cells detectable up to a year after infusion. It is hoped that upcoming early phase clinical trials in patients with CD will inform safety, dosing and Treg kinetics and dispersal allowing further development of a novel therapeutic option in this hard-to-treat population.
